# Defining and categorizing outcomes of Moral Case Deliberation (MCD): concept mapping with experienced MCD participants

**DOI:** 10.1186/s12910-018-0324-z

**Published:** 2018-11-19

**Authors:** Janine C. de Snoo-Trimp, Bert Molewijk, Henrica C. W. de Vet

**Affiliations:** 1 Department of Medical Humanities, Amsterdam University Medical Centers location VUmc, Amsterdam, the Netherlands; 2 Center for Medical Ethics, Institute of Health and Society, Faculty of Medicine, University of Oslo, Oslo, Norway; 3 Department of Epidemiology & Biostatistics, Amsterdam University Medical Centers location VUmc, Amsterdam, the Netherlands

**Keywords:** Clinical ethics support, Moral case deliberation, Concept mapping, Evaluation, Outcomes

## Abstract

**Background:**

To support healthcare professionals in dealing with ethically difficult situations, Clinical Ethics Support (CES) services like Moral Case Deliberation (MCD) are increasingly implemented. To assess the impact of CES, it is important to evaluate outcomes. Despite general claims about outcomes from MCD experts and some qualitative research, there exists no conceptual analysis of outcomes yet. Therefore, the aim of this study was to systematically define and categorize MCD outcomes. An additional aim was to compare these outcomes with the outcomes in the Euro-MCD Instrument from 2014, to further validate this Instrument.

**Methods:**

The concept mapping method was used and involves qualitative and quantitative steps including brainstorming, individual structuring, computation of concept maps (by principal component analysis and cluster analysis), group interpretation and utilization. In total, 12 experienced MCD participants from a variety of professional backgrounds participated in two sessions.

**Results:**

The focus group brainstorm resulted in a list of 85 possible MCD outcomes, of which a point map and concept maps were constructed. After a thorough discussion of each cluster, final consensus was reached on the names and position of 8 clusters of MCD outcomes: 1) Organisation and Policy; 2) Team development; 3) Personal development focused on the Other Person; 4) Personal development as Professional, focused on Skills; 5) Personal development as Professional, focused on Knowledge; 6) Personal development as an Individual; 7) Perception and Connection; and 8) Concrete action.

**Conclusions:**

This study explored and categorized MCD outcomes in a concept mapping focus group. When comparing the results with the Euro-MCD Instrument, our study confirms that outcomes of MCD can be categorized in clusters referring to the organisational level, team development, personal development (both as an individual and a professional) and the concrete case-level. In developing CES evaluation tools, it is important to be explicit if an outcome refers to the individual or the team, to knowledge or skills, to the organisation or the specific case. The findings will be used in the further validation of the Euro-MCD Instrument. The current study further contributes to the field of evaluating CES in general and defining outcomes of MCD in particular.

**Electronic supplementary material:**

The online version of this article (10.1186/s12910-018-0324-z) contains supplementary material, which is available to authorized users.

## Background

Healthcare professionals are confronted with ethically difficult situations every day. When aiming for good care, they face moral questions like: What is good care in this situation? Who should determine what good care is? How to deal with different viewpoints on good care? Clinical Ethics Support (CES) aims to help healthcare professionals in dealing with these situations. CES can take many forms, one of which is Moral Case Deliberation^1^ (MCD) [1]. In an MCD, healthcare professionals jointly engage in a dialogue about a situation from their own clinical practice which they experienced as ethically challenging, under supervision of a trained facilitator and using a structured conversation method [1, 2].

While the presence of and need for CES services like MCD is increasing [3], empirical research about their quality and their impact on healthcare has been scarce [4–6]. MCD evaluation research focusing on outcomes is important in order to know whether MCD actually *supports* healthcare professionals in dealing with ethical challenges, and if so, in which way. Furthermore, evaluation results can be used for the training of future MCD facilitators [2] and the implementation of MCD [7]. Finally, evaluation results can inform the normative discussion what impact of MCD *should* be (e.g. which outcomes are appropriate and which are not?).

Recent evaluation research in the field of MCD showed positive results regarding the participant’s need for and their satisfaction with the CES services [6, 8–14]. However, despite increasing attention for evaluating MCD itself, only few studies systematically evaluated the outcomes of MCD sessions. For instance, Lillemoen and Pedersen [11] have evaluated ethics reflection groups by using qualitative research methods in which they asked ‘What is the significance of ethics reflection groups on health care professionals’ practice?’. Recently, Hem et al. [15] have also studied the significance of ethics reflection groups in mental healthcare. Weidema and colleagues [14] tried to measure the impact of MCD (described as ‘harvest’) by asking MCD participants to answer the question ‘What changes would you apply to your practice after this session?’. In all studies, they found that the ethics reflection groups or MCDs positively influenced the cooperation among colleagues and made professionals better able to deal with ethical challenges in everyday practice [11, 14, 15]. Questions about the significance and harvest of MCD are relevant, but it remains unclear what these terms exactly entail. For future CES and MCD evaluation it is important to systematically develop clear conceptual categories which can be used within various European settings. This leads to questions such as: How should we define outcomes of MCD sessions and how should we conceptualize different categories of MCD outcomes? In literature, there exists no systematic conceptual analysis of MCD outcomes yet.

Since MCD aims to support healthcare professionals and because MCD focuses on the experiences and perspectives of the MCD participants themselves, it is important to involve actual MCD participants in empirical research about possible MCD outcomes. This does not imply that outcomes that are reported, experienced or valued by MCD participants should automatically become the *normative* goals of MCD. Yet, experiences from MCD participants play an important role in defining possible outcomes since they are the actual users of MCD. They can, based on their experiences with MCD, help in reporting which outcomes they experienced and how they think MCD outcomes are connected to one another.

In order to contribute to the professionalization of both MCD itself as well as the evaluation research of MCD, a European research project on outcomes of MCD has been started. In this project, an MCD evaluation tool called the ‘Euro-MCD Instrument’ has been developed [16]. The Euro-MCD Instrument is a questionnaire for actual MCD participants and lists 26 possible outcomes of MCD, divided over 6 domains (Emotional Support; Moral Reflexivity; Moral Attitude; Collaboration; Concrete Results and Impact on Organisational Level) [16]. MCD participants rate the ‘perceived importance’ of these 26 outcomes of MCD both before and after participation in a series of 4 to 8 MCD sessions. They also rate whether they actually ‘experienced these outcomes within the MCD sessions and in daily practice’ [16, 17]. The list of 26 possible outcomes was established by a European research team (of which ACM is author of this paper); based on a combination of explorative literature review and a Delphi panel with European CES and MCD experts [16]. Currently, a large European field study is carried out to collect empirical data and validate the Euro-MCD instrument [17].

Although the Euro-MCD Instrument asks for the input of MCD participants regarding their perceived importance and experience of MCD outcomes, MCD participants did not play a role in the development of the instrument. Therefore, it is yet unknown what outcomes they would mention themselves and how they would categorize these outcomes. In order to explore and define possible MCD outcomes as mentioned by experienced MCD participants and to form an evidence-based categorization of these outcomes, the current study was performed. The ultimate goal of the larger Euro-MCD field study (of which this study was part of) was to improve and further validate the current Euro-MCD Instrument in order to professionalize future CES and MCD evaluation research. Therefore, we compared the findings with the original items of the Euro-MCD Instrument in the Discussion section.

## Methods

### Steps of concept mapping

**Fig. 1:**
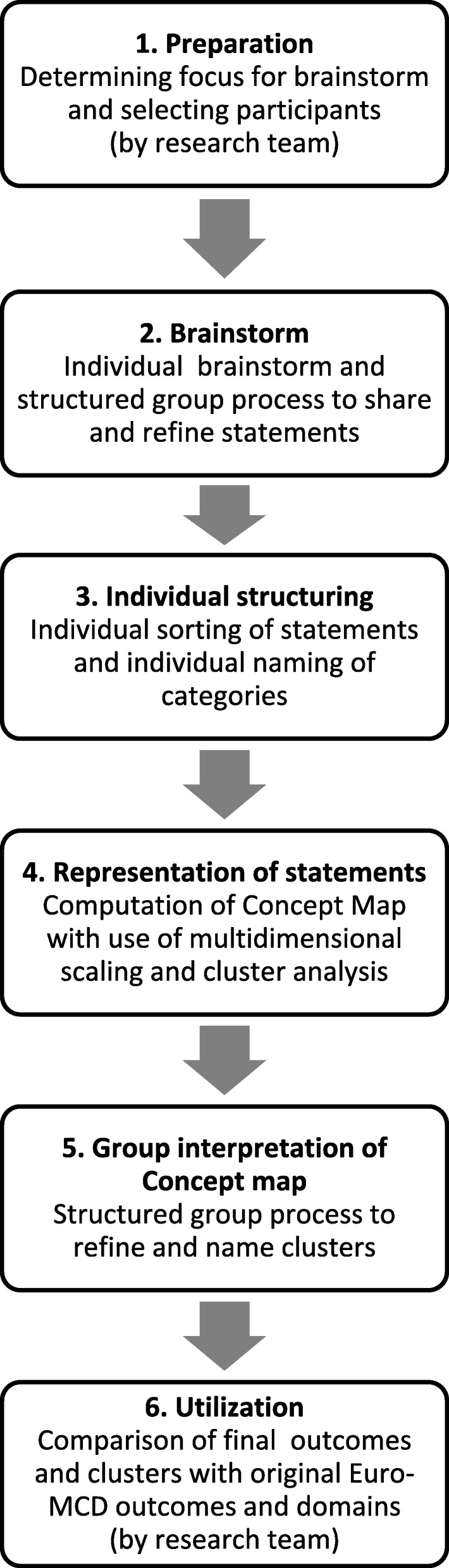
Steps Concept Mapping. Modified from Kane and Trochim [19]

Concept mapping is a methodology for conceptualization and categorization of a complex topic [18, 19]. A ‘collaborative, participatory process’ takes place in a focus group, consisting of 6 qualitative and quantitative steps (using visualization techniques), as shown in Fig. 1 [19]. Qualitative steps include brainstorming, structuring and interpretation while quantitative steps include the computation of a concept map. Focus group members are involved from step 2–5; from the initial brainstorming until the analysis of the final concept map, including the naming of the final categories. These steps took place during two sessions. The final result is a graphic representation of ideas (the concept map), build, understood and clarified by all focus group members.

We will now describe how we performed these six steps.

#### Preparation: Focus and participants

In the preparation phase, the research team determined the focus for the concept mapping by articulating the *focus statement* [19]: ‘What are possible outcomes of Moral Case Deliberation?’ We aimed to exclude both too abstract outcomes and too concrete outcomes, (e.g. about healthcare in general, or about a specific case). One member of our research team (ACM) was the facilitator.

The first step further consisted of selecting the participants. The aim in our study was to form a group with experienced MCD participants, preferably from many various settings where MCD is practiced and from diverse professional backgrounds. They were recruited by JDST in two ways: 1) by inviting all trainees of the current Dutch national training program for becoming a MCD facilitator, and 2) by inviting members of the national network of certified and experienced MCD facilitators. Trainees needed to have extensive experience with participating in MCD sessions before the start of the training program [2]. At the time of the focus group, the MCD facilitator training program was almost finished, therefore, the trainees were experienced and well-known with MCD.

#### Brainstorm

The second step formed the start of the actual concept mapping process. The aim was to generate a list of statements that ‘ideally, will represent the entire conceptual domain for the topic of interest’ [19].

During the first meeting, the facilitator (ACM) firstly asked focus group members to take 10–15 min to think of possible MCD outcomes individually. He then asked them to present their statements one by one. The statements were directly entered into a word processing program by a research team member (JDST) and projected on a large screen. By this, all focus group members could see the statements and discuss the distinctiveness and clarity of them, as it was important that everyone understood and agreed on the generation of statements (i.e. that statements were not on the screen already or too vague to understand).

After the round in which participants shared their own statements, one member of our research team (JDST) presented additional MCD outcomes from the Euro-MCD study that were not yet included in the list made during the focus group. These outcomes came from the list of 26 items of the Euro-MCD Instrument and outcomes mentioned by Dutch respondents to the open question in the instrument asking for possible MCD outcomes [16]. The characteristics of these respondents are shown in Additional file 1 JdST only presented outcomes that were not yet mentioned, and the focus group had the final say in adding the suggested outcome to the list, depending on their distinctiveness and clarity. By this, we aimed to get as rich data as possible.

The step of sharing and refining would finally result in a list of unique and clear statements. The facilitator (ACM) aimed to generate no more than 100 statements, in order to avoid practical difficulties with the next steps of the concept mapping process [19].

#### Individual structuring

In the third phase, focus group members were asked by the facilitator (ACM) as an individual task to structure the statements into piles “in a way that makes sense to you” [19] and to give a unique name to each pile (without communicating with other focus group members). They were not allowed 1) to make a single pile including all statements; 2) to make as many piles as statements; and 3) to make a pile ‘remaining/other statements’. Next, if focus group members wanted to make a pile including only one statement, they should still give this pile a unique name.

In our study, the research team printed all outcomes from the brainstorm on separate cards, and gave everyone the complete set of cards. After the individual sorting task, we collected all piles including names with paper clips and put them into envelopes; one envelope per focus group member.

#### Representation of statements

The fourth phase consisted of the computation of concept maps based on the thematic clusters (piles) made by all focus group members individually. This was done in-between the two sessions, in which the research team (JDST and HCWDV) entered the piles from all members into a concept mapping software program, Ariadne [20]. This software program examines the frequency of any two statements occurring in the same pile. The number of similarities are then put into an aggregated group matrix, on which a Principal Component Analysis (PCA) was performed, resulting in a point map. PCA translates the correlations between statements into distances and coordinates on the point map [18]. Statements that were put in the same pile by many members would be located close to each other on the map; statements that were not put together in the previous phase or only by a few members would be located on the map with more distance. The software ignores the names given to the piles by the focus group members. Next, the software performs cluster analysis to make a cluster map, in which closely-related statements are represented as clusters. The number of clusters could be determined by the researchers and as such, different cluster maps could be presented. The research team chose a concept map with an ample number of clusters to take as starting point for the second session of the focus group. This concept map was chosen because it leaves room for focus group members to still merge clusters.

#### Group interpretation of concept map

A month after the first session, the second session was organized. In the fifth step, the concept map was discussed and interpreted with the focus group. Firstly, the point map and concept map were presented to the focus group. Secondly, focus group members were asked by the facilitator (ACM) to take some time to read all statements and explore their place on the map, and to write down possible names for each cluster. Thirdly, the focus group engaged in a dialogue about the map. Cluster by cluster, members presented their thoughts and names and tried to reach agreement on the final name of each cluster. This phase could also include refining clusters by changing the position of separate statements.

In our study, the research team showed the concept map on the screen and on paper for each focus group member. The facilitator (ACM) aimed to reach consensus among the participants on the final number and naming of clusters, by discussing them cluster by cluster. If no consensus could be achieved, the majority decided, but other research team members (JDST and HCWDV) made a note of this. The facilitator of the focus group (ACM) invited members repeatedly to express their opinions, in particular if they disagreed with others regarding the number and naming of the clusters. Those who still disagreed after discussion could withdraw from further discussion on the naming or content of that specific cluster. All disagreements and withdrawals from discussions on clusters were reported by the research team.

#### Utilization

The final concept map can be used for planning and evaluation purposes. With regard to evaluation, it can be seen as a guide for measurement development, or as a framework for examining patterns of outcomes as it provides a clear overview of statements and their mutual relations [19]. For instance, in developing a questionnaire, the concept map can be used as basis for building domains and topics of questions.

The research team compared the final statements and clusters of the concept map with the original outcomes and domains of the Euro-MCD Instrument. This will be described in the Discussion section.

### Research ethics

Conform the ethical principles for medical research as stated in the Declaration of Helsinki [21], all focus group members signed an informed consent form describing the purpose of the study and the way data would be collected and analyzed. All members were informed that participation was voluntary and that they could withdraw from participation in this study at any moment and without giving any reason. The collected data from focus group members was anonymized and inserted into the concept mapping software with codes not able to trace back to individual members.

## Results

In total, 12 experienced MCD participants, from a variety of professional backgrounds, took part in the concept mapping focus group. The characteristics of them are presented in Table 1. Almost all of them were MCD facilitators with an average of 5 year experience. They were all present in both sessions of the focus group, lasting 2 h each.

**Table 1:** Characteristics Focus group members (*N* = 12)

Female/male	8/4
Mean age (range)	53 (31–64)
Mean working experience (range)	15 (2–30)
Facilitator of MCD/in training	9/3
Mean experience as facilitator (range)	5 (0–10)
Profession	1 Nurse
	1 Physician
	2 Spiritual Caregivers
	3 Coaches
	2 Researchers/Teachers
	1 Manager
	1 Head of Ethics Committee
	1 Quality Officer

Settings where they facilitate MCDs: *Elderly care, nursing homes, hospitals, care for mentally disabled, psychiatry, science, prisons, municipality, business, education*

In the first session, the brainstorm phase resulted in a list of 85 unique and clear statements, according to the focus group members, as presented in Additional file 2. From these 85, 68 came from the focus group members themselves. The other 17 statements came from a list of 19 statements that was presented by the research team after the focus group had completed their own brainstorm phase. From these 17 statements, 11 came from the original Euro-MCD Instrument and 6 statements came from open answers of Dutch respondents to the Euro-MCD Instrument in an earlier study (their characteristics are shown in Additional file 1). The 17 statements were adopted based on consensus among the focus group members, as they had the final say in adding the statement with regard to its distinctiveness and clarity.

**Fig. 2:**
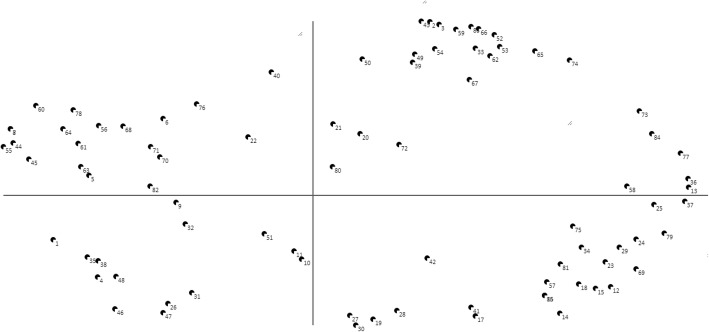
Point map based on the individual categorizations of focus group members. Each point represents a statement from the brainstorm phase. Statements that are put in the same pile (during the previous step of individual sorting) by many members will be located close to each other on the map; statements that are not put together in the same pile or only by a few focus group members will be located on the map with more distance

All focus group members finished the step of individual structuring. They sorted the statements into 5–20 piles. Figure 2 shows the resulting point map, in which all 85 statements are represented. Their position is dependent on their relations with other statements For example, the statements ‘A concrete plan of action, a or b’ (no. 10) and ‘Plan of action on how to deal with damage’ (no. 11) are located very close to each other on the point map since these statements were put into the same pile by 11 out of 12 focus group members.

**Fig. 3:**
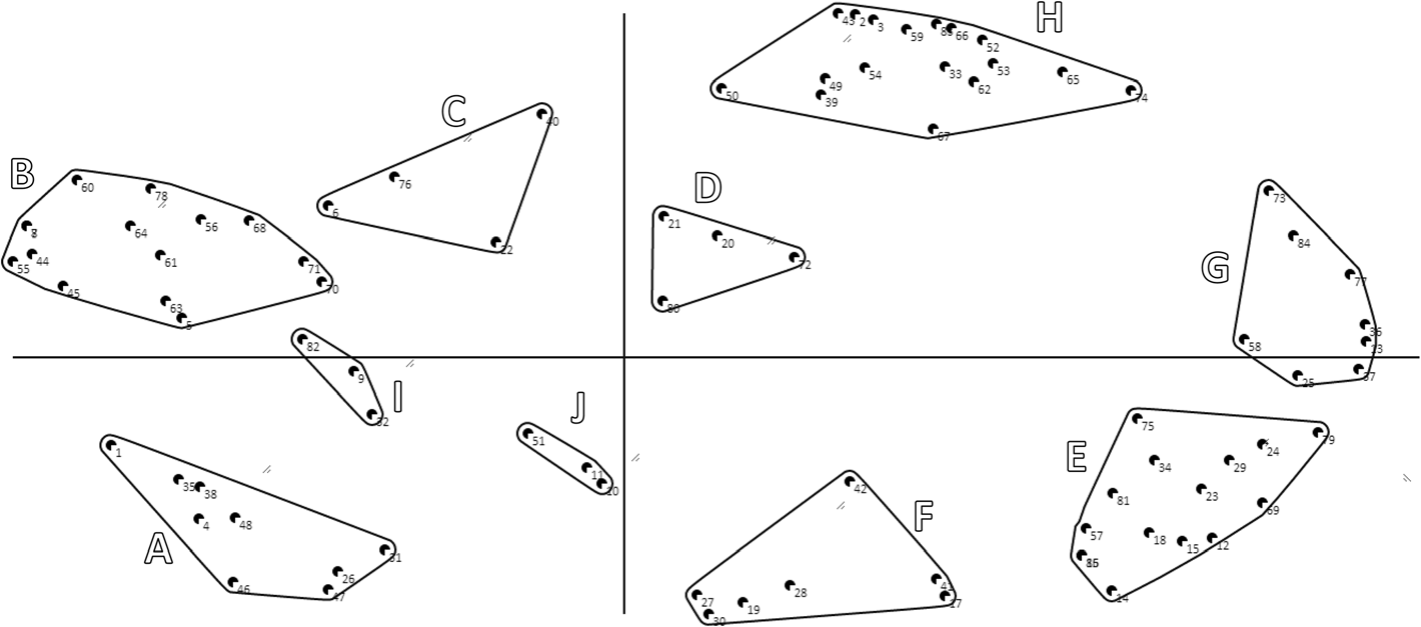
Concept map with 10 clusters (**a**-**j**) of MCD outcomes, based on individual categorizations of focus group members. Each point represents a statement from the brainstorm phase. Statements that are put in the same pile (during the previous step of individual sorting) by many members will be located close to each other on the map; statements that are not put together in the same pile or only by a few focus group members will be located on the map with more distance. The contoured forms represent clusters of closely-related statements, as made by the concept mapping software program (Ariadne©). The letters A to J refer to the 10 clusters in the order of how they were presented for discussion during the second session of the focus group

The cluster analysis provided the possibility to construct 2 to 12 clusters, which were discussed with the research team (JDST, HCWDV, ACM). The concept map of 10 clusters was chosen as starting point for the next session of the focus group (see Fig. 3) because all clusters had at least 3 items and the more clusters a map has, the more possibilities participants have for merging clusters, which gave us more nuanced conceptual information.

**Fig. 4:**
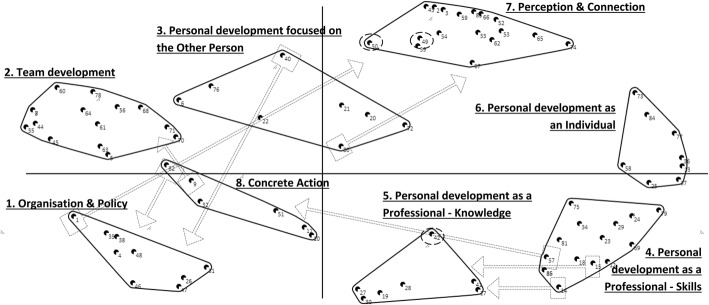
Concept map with 8 final clusters, including names and item replacements, based on group interpretation. Each point represents a statement from the brainstorm phase. Statements that are put in the same pile (during the previous step of individual sorting) by many members will be located close to each other on the map; statements that are not put together in the same pile or only by a few focus group members will be located on the map with more distance. The contoured forms represent clusters of closely-related statements, as made by the concept mapping software program (Ariadne©). The text next to each cluster represents the final name given to that cluster by the focus group. Statements in dotted boxes were discussed during the focus group and considered to fit better in another cluster; the striped arrow shows into which cluster it was reallocated. Statements circled by dashed lines were discussed during the focus group but not replaced (see also Table 2)

The concept map with 10 clusters was discussed with the focus group in the second session, with the aim to find consensus on the number and naming of the clusters, as described in the Methods section. Focus group members were explicitly asked to express their (dis)agreement with categorizations and possible names of clusters. This was the case during the categorization or specification of a few clusters, which will be described to more detail below. In the end, after some statement replacements, consensus was achieved on the names and position of all statements in 8 final clusters. The final concept map is presented in Fig. 4, where the names of the clusters (defined by the focus group members) are added to the clusters of statements as described earlier. The striped arrows show which items have been replaced to another cluster, based on the group discussion, and the dashed circled statements represent statements that were discussed but not replaced. The final categorization of clusters and statements is presented in Table 2. We will now describe the 8 final clusters on which the group reached consensus in more detail, according to the order they were discussed in the focus group.

**Table 2:** Final clusters of MCD outcomes, including items and comparison with Euro-MCD items and domains

Clusters	# Items *Original items from Euro-MCD Instrument		*Comparable to which Euro-MCD item according to research team; empty = no comparable item found* *(for all items except the 11 Euro-MCD items that were explicitly added in the focus group)*	*From which Euro-MCD Domain*
1. Organisation and Policy *(A in* Fig. 3*)*	4	Support to proceed in a particular direction	Enables to decide on concrete actions	*Concrete Results*
	26	Identify relevant themes	Identify core ethical question	*Moral Reflexivity*
	31	Clarify what good care entails	-	
	35	Contribute to organisational change or cultural shift	Contribute to development of practice/policies	*Organisational Level*
	38	Support in the development of new products and services	Contribute to development of practice/policies	*Organisational Level*
	40	Less absence due to sickness	-	
	46	Prevention of similar case/event in the future	Become aware of recurring ethical situations	*Organisational Level*
	47	Anticipate, show restraint in similar case/event	Become aware of recurring ethical situations	*Organisational Level*
	48	Initiate formulation of policy	Contribute to development of practice/policies	*Organisational Level*
	82	Better quality of work	-	
2. Team development *(B. in* Fig. 3*)*	5	Accepting an outcome	-	
	7	Unity in teams/increased mutual cohesion	-	
	8	Team spirit/sense of belonging to group	-	
	9	Difficult themes can become subject of discussion	-	
	44	Team continues with method of examination as in MCD	-	
	45	Team jointly determines team values	-	
	55	Less hierarchical interaction	-	
	56	Gentler communication	-	
	60	Get to know each other better	Better mutual understanding	*Enhanced Collaboration*
	61	Enhance professional identity	Better understanding of being good professional	*Moral Attitude*
	63	Commitment to the organisation	-	
	64	Help each other more readily	-	
	68	More open communication*	***Not applicable – original Euro-MCD item***	*Enhanced Collaboration*
	70	Greater opportunity for everyone to have their say*	***Not applicable – original Euro-MCD item***	*Enhanced Collaboration*
	71	Share difficult emotions and thoughts*	***Not applicable – original Euro-MCD item***	*Emotional Support*
	78	Mutual respect*	***Not applicable – original Euro-MCD item***	*Enhanced Collaboration*
3. Personal development focused on the other person *(C and D in* Fig. 3*)*	6	Curiosity about the other person	-	
	20	More sensitive to the perspective of another person	See the situation from different perspectives	*Moral Reflexivity*
	21	Accepting other person’s perspective	-	
	22	Appreciate other person’s perspective	-	
	72	I listen more seriously to others’ opinions*	***Not applicable – original Euro-MCD item***	*Moral Attitude*
	76	Manage disagreements more constructively*	***Not applicable – original Euro-MCD item***	*Enhanced Collaboration*
4. Personal development as a professional, focused on Skills *(E in* Fig. 3*)*	14	Learn what the relevant norms and values are	-	
	15	Knowledge of ethical concepts	Enhanced understanding of ethical theories	*Moral Reflexivity*
	17	Understanding which values and norms are in conflict with each other	-	
	19	Understanding of diversity of norms and values, interpersonal	-	
	24	Postpone personal judgments about situations	-	
	27	Identify recurring norms and values in particular themes	Identify core ethical question	*Moral Reflexivity*
	28	Identify how moral issues are dealt with	-	
	30	Tools to reflect on moral dilemmas	Skills to analyze	*Moral Reflexivity*
	41	Clarity about what the issue is and what is at stake	Identify core ethical question	*Moral Reflexivity*
	42	More awareness of unequal balance of power	-	
5. Personal development as a professional, focused on Knowledge *(F in* Fig. 3*)*	12	Dilemma awareness, increased moral sensitivity	Identify core ethical question	*Moral Reflexivity*
	23	Postpone personal judgments about individuals	More awareness of preconceived notions	*Moral Attitude*
	29	Reflexive skills	Skills to analyze	*Moral Reflexivity*
	34	Master at asking questions	-	
	69	Increased awareness of complexity of situation*	***Not applicable – original Euro-MCD item***	*Moral Reflexivity*
	75	Examine practice/policies critically*	***Not applicable – original Euro-MCD item***	*Organisational Level*
	79	Change your mind	-	
	81	Take a step back to look at problem from a distance	-	
	85	Understanding of role-related quality of norms and values	-	
6. Personal development as an individual *(G in* Fig. 3*)*	13	More awareness of personal judgment	More awareness of preconceived notions	*Moral Attitude*
	25	Examine personal judgment	-	
	36	Reduce blind spots	-	
	37	Increase awareness of your blind spots	-	
	58	Creative thinking	-	
	73	Increased awareness of own emotions*	***Not applicable – original Euro-MCD item***	*Emotional Support*
	77	Gain more clarity about own responsibility*	***Not applicable – original Euro-MCD item***	*Moral Attitude*
	84	Become more honest	-	
7. Perception and Connection *(H in* Fig. 3*)*	1	Substantiate decision made by staff member	Consensus in how to manage the situation	*Concrete Results*
	2	Feel you do not have to deal with the problem alone	-	
	3	Support	-	
	33	Relieves stress for case presenter	Better manage stress from the situation	*Emotional support*
	39	Delight in astonishment about differences	-	
	43	Person presenting the case feels s/he is heard	-	
	49	Disappointment about outcome	-	
	50	Sense of wasting time	-	
	52	Recognition brings sense of relief	-	
	53	Sense of relief	-	
	54	Fewer psychological complaints	-	
	59	Enjoy your work more	-	
	62	Increased motivation regarding work	-	
	65	Feel enriched by unexpected new insights	-	
	66	You feel taken care of, nurturing for your inner self	-	
	67	Be able to move on	-	
	74	Strengthened self-confidence*	***Not applicable – original Euro-MCD item***	*Emotional Support*
	80	Confirmation of having made the right decision	-	
	83	To feel safe	Feel more secure to express doubts/Courage to express doubts or uncertainty	*Emotional Support/Moral Attitude*
8. Concrete action *(I and J in* Fig. 3*)*	10	A concrete plan of action, a or b	Enables to decide on concrete actions	*Concrete Results*
	11	Plan of action on how to deal with damage	-	
	32	Look for the answer for the client/central person in the case	-	
	51	More awareness how personal values influence working together	-	
	57	Increase range of action	Find more courses of action	*Concrete Results*

#The numbers of the items correspond to the numbering in Fig. 2

*Original items from Euro-MCD Instrument

### Cluster 1 – Organisation and policy

The first discussed cluster was cluster A in Fig. 3. It was considered as a diverse cluster according to the focus group members. The statements refer to the content of care, development of policies, vision and the organisation as such. The name ‘Organisation and Policy’ was suggested and agreed upon. Some members shortly discussed whether this name is sufficiently linked to MCD, but the group decided that because the outcomes are defined as outcomes of MCD, the cluster name intrinsically refers to MCD. Regarding the statements, item 1 (‘Substantiate decision made by staff member’) was not perceived as linked to the cluster name, therefore it was removed to cluster 7 (later named as ‘Perception and Connection’).

### Cluster 2 – Team development

The focus group members quickly reached consensus on the name of the second cluster (B in Fig. 3): ‘Team development’. According to the focus group members, statements within this cluster refer all to the development as a team and none needed to be replaced or reconsidered.

### Cluster 3 – Personal development focused on the other person

The majority of the group proposed to merge clusters C and D (Fig. 3). Some also wanted to integrate it in cluster 2 ‘Team development’, but according to others this should form a separate cluster as it is about individual development, instead of group development, but it is linked to the team. Therefore the name ‘Personal development focused on the Other Person’ was suggested. Two focus group members did not think cluster D should be a cluster at all, as its statements would better fit in other clusters. In the end, the majority agreed on merging cluster C and D into one cluster and to remove several statements from cluster D to other clusters. This is shown in Fig. 4.

### Cluster 4 and 5– Personal development as a professional, focused on skills (4) and knowledge (5)

These clusters correspond with cluster E (4) and F (5) in Fig. 3. During the focus group, some members disagreed on the question if cluster E and F should be merged or not. A possible name for the merged cluster was suggested: ‘ethical awareness’. But after an extensive discussion, it was decided that although both clusters refer to personal development as a professional, they should remain separate clusters because cluster 4 (E) emphasizes the skills and cluster 5 (F) emphasizes the knowledge within personal development. One focus group member did not agree upon this decision. Some statements were moved from E to F and vice-versa, and one statement (57, ‘Increase range of action’) was moved to cluster 8 (later named as ‘Concrete action’).

### Cluster 6 – Personal development as an individual

The statements in cluster 6 (G in Fig. 3) involve self-reflection, self-insight and personal development in general, according to the focus group members. They considered whether or not it should be merged with cluster 5 (‘Personal development as a professional – skills’), but statements were not seen as necessarily linked to professional tasks. One focus group member said that he had developed several skills – through participation in MCD – which he also uses in the non-professional context. Consensus among all members was found on the following name for this separate cluster: Personal development as an Individual.

### Cluster 7 – Perception and connection

The focus group interpreted the statements within cluster 7 (H in Fig. 3) as emotions, feelings, support and contact with colleagues. They shortly discussed whether the two ‘negative’ statements really belong in this cluster: ‘Disappointment about outcome’ (no. 49) and ‘Sense of wasting time’ (no. 50). The cluster name ‘Perception and Connection’ was suggested by a focus group member and the group eagerly agreed with this name, as this name leaves room for ‘perceiving’ both positive as well as negative outcomes.

### Cluster 8 – Concrete action

This cluster refers to clusters I and J in Fig. 3. According to some focus group members, these clusters should be merged, but others perceived them as separate clusters. Two members thought the clusters should not exist at all since these were too small with too diverse statements, and they did not contribute to the further discussion about the naming of this cluster (. The discussion with the remaining members was mainly about the question: do the statements refer to skills or to acting? They finally agreed on the latter; the statements are about concrete actions and practical acting. The majority of the focus group therefore decided to name the cluster ‘Concrete action’. According to some members, this cluster is highly important as outcomes referring to choices, decisions and practical acting did not yet have a clear place in the focus group. Several statements were moved to other clusters (see Fig. 4 and Table 2).

## Discussion

In this study, experienced MCD participants from a broad range of settings where MCD is practiced, explored possible MCD outcomes using the qualitative and quantitative method of concept mapping [18]. The concept mapping focus group with 12 members provided a list of 85 possible outcomes. In the end, a clear categorization of 8 clusters that comprehends 85 possible outcomes was achieved: 1) Organisation and Policy; 2) Team development; 3) Personal development focused on the Other Person; 4) Personal development as Professional, focused on Skills; 5) Personal development as Professional, focused on Knowledge; 6) Personal development as an Individual; 7) Perception and Connection; and 8) Concrete action.

### Reflection on focus group process

The focus group members came from a variety of professional backgrounds and had broad experiences with MCD participation in different settings (even outside healthcare). Due to this, they brought a large variety of statements into the brainstorm phase and at the same time were able to critically analyze the final list of statements. During the step in which they had to make piles of the statements, the number of piles differed. One focus group member distinguished only 2 thematic clusters covering all 85 outcomes while other distinguished up to 20 thematic clusters. A step for step dialogue in which they had the possibility to explain how they thought about the differentiation of clusters, relatively easily led to an agreement about how many clusters there should be. Furthermore, a surprising finding was that with regard to some clusters (Organisation and Policy, Team development, Personal development as an Individual and Perception and Connection), consensus was easily reached, possibly indicating that the cluster was recognizable as a specific theme and probably clearly enough constructed. However, the discussion about the other clusters took a while, and some focus group members did not contribute to the formulation of cluster names as they did not support the positioning of some clusters. Nevertheless, in the end, all focus group members agreed on the final naming and categorizing of the 8 clusters, despite the minor disagreements during the cluster discussions.

When exploratively reviewing literature, we found some (but not all) of these clusters as well. For instance, in the focus group study of Hem et al. [15], participants of ethics reflection groups (which is similar to MCD) described that they experienced an increased awareness of ethical issues, ‘professional development’ and better collaboration among their colleagues, in which we see a clear link with two clusters in our study, namely Team development and Personal development as a Professional. A better team collaboration and the impact on personal development have also been suggested by other studies [11, 13, 14]. Next, in the study of Seekles et al. [12], professionals working in the Dutch Health Care Inspectorate reported to feel more secure after participating in MCDs, which refers to several statements in the cluster Perception and Connection in our study. Furthermore, Lillemoen and Pedersen [11] reported how participation in ethics reflection groups contributed to ‘important changes in practice’, for example by improving their attitude towards and cooperation with patients and their relatives. This impact on concrete practice shows a link with the cluster Concrete Actions in our study. Thus, the clusters found in our study can to some extent be confirmed by other studies. However, all of these studies did not explicitly focus on the naming and meaning of the clusters and the mutual relationship between statements within clusters, which was systematically explored in the current study. Especially with regards developing tools to evaluate MCD outcomes, our findings are relevant to operationalize and concretely define what (categories of) MCD outcomes mean according to a heterogeneous group of experienced MCD participants.

### Comparing focus group-clusters with euro-MCD domains

According to the second aim of this paper and the sixth step of concept mapping [19] and in light of the ultimate goal of the Euro-MCD field study, we would like to compare the outcomes and clusters as defined in the two concept mapping focus group sessions with outcomes from the Euro-MCD study [16, 17]. During the focus group, 15 out of the 26 items in the Euro-MCD Instrument were already spontaneously mentioned in the brainstorm phase, and 11 of these 26 items were added afterwards (i.e. when presented as possible outcomes, the focus group members approved these 11 Euro-MCD outcomes as relevant). As shown in Table 2, these 11 added outcomes came from different Euro-MCD domains, but it is remarkable that especially items from the domain of Collaboration were added at that moment; hence, they were not yet mentioned in the preceding brainstorm phase. The fact that almost no items from the domains of Concrete Results, Moral Reflexivity and Moral Attitude were added, means that these or similar outcomes were already brought up during the brainstorm phase, which might point to a tendency of the focus group members to think of outcomes linked to these domains.

When comparing the final cluster names with the names of the Euro-MCD domains, several links can be made: Concrete Action with Concrete Results; Organisation and Policy with Impact on Organisational Level; Team development with Collaboration. Furthermore, when looking at the Euro-MCD domain Emotional Support, we see a link with our cluster Perception and Connection, as both include feelings and emotions like ‘self-confidence’, ‘managing stress’ and ‘feeling secure’. Finally, the Euro-MCD domains Moral Reflexivity and Moral Attitude can be compared with the clusters about personal development (clusters 3–6), as they all include outcomes referring to self-reflection, like ‘I gain more clarity about my own responsibility in the ethically difficult situations’ and ‘Increases my awareness of the complexity of the situation’. Fortunately, we can therefore conclude that the original categorization of MCD outcomes by MCD experts in the Euro-MCD Instrument can be confirmed to some extent, despite the fact that their categorization was not yet based on empirical data at that time [16].

However, several differences can be found as well when comparing the Euro-MCD domains with the clusters of the concept map. Firstly, the Euro-MCD domains Emotional Support and Collaboration seem to be reflected in more than 2 focus group-clusters, namely Team development, Personal development focused on the Other Person and Perception and Connection. Secondly, the Euro-MCD domains Moral Reflexivity and Moral Attitude cannot be recognized easily in the focus group-clusters, since terms like ‘reflexivity’ or ‘attitude’ were not used. These domains are about analytic skills, awareness and understanding of ethically difficult situations. Yet, the focus group members made a distinction between skills and knowledge in their separate clusters about personal development as a professional (4 and 5). To conclude, we can say that the focus group members defined additional and more detailed categories of outcomes that match with outcomes from the Euro-MCD domains Moral Reflexivity and Moral Attitude, namely based on whether the outcome was about personal or professional development, about oneself or directed to the other, and about skills or about knowledge. This resulted in 4 separate clusters (3–6) for personal and professional development, and skills and knowledge. This difference in nuances might be explained by the fact that the definition of the Euro-MCD domains of Moral Reflexivity and Moral Attitude was based on theory, literature and the opinion of MCD experts [16], while the naming of the 4 focus group clusters about personal development in the current study was only based on the practical experience of actual MCD participants. In our opinion, this might show the added value of both using the method of concept mapping and giving voice to the concrete users of MCD focusing on their experiences with participating in MCD sessions.

### Negative outcomes of MCD

An interesting difference between this study and the Euro-MCD Instrument is the formulation and position of possible negative outcomes (‘Disappointment about outcome’ and ‘Sense of wasting time’) in the focus group cluster Perceiving and Connecting, while the Euro-MCD domains only contain positively formulated outcomes. The formulation of these two negative outcomes was a surprising finding in our study, although the number of two might be a quite low number. We think and literature shows that MCD might cause negative outcomes as well, like frustrations about the lack of solutions [22] or not experiencing changes in daily work [10, 14]. A reason for this could be that defining possible outcomes of MCD is closely linked to how participants experienced the MCDs they participated in. The focus group members in our study all had extensive experience with MCD and did thus not base their thoughts on only one positive (or negative) MCD. It is important for future research on outcomes of MCD to make sure if outcomes of MCD really involve *outcomes* and not the *process* of MCDs themselves. Future qualitative studies should investigate what kind of negative outcomes MCD participants report, whether they refer to literally negative or harmful outcomes or a lack of expected positive outcomes, and in which way they are related to MCD as such. This is important in order to avoid a bias in presenting (only positive) MCD evaluation results. Furthermore, negative MCD outcomes could be helpful in improving, adjusting or not using MCD as ethics support mechanism. Furthermore, we should reflect upon the question whether we should pay attention to negative MCD outcomes in the further validation of the Euro-MCD Instrument.

### Strengths

One of the strengths of this study was the fact that the concept mapping procedure consisted of structured and systematic conceptual-analytical steps in which qualitative and quantitative measures were integrated within a reflective open dialogue. A main strength of our study was the composition of the focus group: members came from various professional backgrounds, in diverse settings of MCD, both inside and outside healthcare, and were all very experienced as participants in MCD. As such, they were no specific ‘experts’ in evaluation research, instrument development, but people from a broad range of settings were MCD is practiced. They were all present and actively involved in both sessions of the focus group, and they all had a critical and analytical yet constructive contribution. This might be caused by the fact that they were experienced MCD participants and thus were used to group sessions with equal participation, a critical dialogue, being open towards different perspectives and letting others express their thoughts. The final concept map with named clusters is a product of the participants themselves, as it was based on statements that they generated in their own words, with extra input from the original Euro-MCD. Furthermore, a methodological strength was that we were able to complement the brainstorm among the focus group members with data from the large Euro-MCD field study as well, in order to get as rich data as possible. Lastly, the focus group members achieved a relatively strong agreement on the final names and categorization of the clusters, resulting in an experience- and consensus-based categorization of MCD outcomes.

### Limitations

Yet, our study has limitations as well. The study contained only one focus group consisting of two sessions in only one country, due to limited time and financial resources. Since we needed experienced MCD participants, the Netherlands was a good candidate for performing this study as MCD is implemented in this country for a long time [3]. It is important to know whether MCD participants from other countries might come up with similar MCD outcomes, not only because of cultural differences but also because of possible differences in how MCD is seen and performed. Thus, in developing instruments to measure outcomes of CES interventions like MCD, data from other countries should also serve as an important basis.

## Conclusions

On the bases of a Dutch focus group study with experienced MCD participants, this paper presented 8 thematic categories for *possible* MCD outcomes with use of the qualitative and quantitative method of concept mapping. Based on these descriptive results, in a future empirical-ethics study, one can start to reflect upon the normative question whether possible outcomes are also desirable outcomes, and for which reason. The study provides valuable lessons for further evaluation research on outcomes of CES services and outcomes on MCD in general. Most importantly, the current study confirms that outcomes of MCD can be categorized in clusters referring to the personal development (both as an individual as well as a professional, including emotions, skills and knowledge), team development, the organisational level and the concrete case-level. Moreover, according to the focus group members in the current study, several (clusters of) outcomes of MCD are related to the cooperation with colleagues, and even feelings and emotions are involved here (in the cluster Perception and Connection). Furthermore, MCD could have an impact on the way participants would act in practice and on policy making at the organisational level. Therefore, in developing measurement tools, it is important to be explicit if an outcome refers to knowledge, or skills, to the person him/herself or to his or her professional role, or to the team, organisation or the specific case.

According to the ultimate goal to further validate the Euro-MCD Instrument of 2014 [16], in the near future, a new version of the Euro-MCD instrument for evaluating MCD outcomes will get presented, being both evidence-based and experience-based. This will make it possible to perform new evaluation studies and get insight in the actual impact of MCD within the daily practice of healthcare professionals in European healthcare.

## Additional files

Additional file 1: Characteristics of Dutch Respondents in Euro-MCD study. This file shows the characteristics of Dutch healthcare professionals who participated in the Euro-MCD study. In this study, they completed a questionnaire (the Euro-MCD Instrument). This questionnaire includes an open question regarding possible outcomes of Moral Case Deliberation, defined and perceived as important by the respondent. The answers on this question were used in the current study. (DOCX 14 kb)

Additional file 2: List of outcomes of Moral Case Deliberation. In this file, the list of 85 outcomes of Moral Case Deliberation is presented, as identified and defined by the participants during the brainstorm phase of the concept mapping focus group. (DOCX 17 kb)

## Abbreviations

CES: Clinical Ethics Support
MCD: Moral Case Deliberation
PCA: Principal Component Analysis

## Footnotes

1 Also described as ‘ethics rounds’ [10, 21]; ‘ethics reflection groups’ [11, 15] and ‘ethics case reflection sessions’ [23].
