# Supplementary material for: Defining and categorizing outcomes of Moral Case Deliberation (MCD): concept mapping with experienced MCD participants
Source: BMC Med Ethics. 2018 Nov 19;19:88. doi: 10.1186/s12910-018-0324-z (PMC6245560; doi:10.1186/s12910-018-0324-z)
Supplement: Supplementary file 1 — Characteristics of Dutch Respondents in Euro-MCD study. This file shows the characteristics of Dutch healthcare professionals who participated in the Euro-MCD study. In this study, they completed a questionnaire (the Euro-MCD Instrument). This questionnaire includes an open question regarding possible outcomes of Moral Case Deliberation, defined and perceived as important by the respondent. The answers on this question were used in the current study. (DOCX 14 kb) [file 12910_2018_324_MOESM1_ESM.docx]

**Additional file 1 – Characteristics of Dutch Respondents in Euro-MCD study**

This file shows the characteristics of Dutch healthcare professionals who participated in the Euro-MCD study. In this study, they completed a questionnaire (the Euro-MCD Instrument). This questionnaire includes an open question regarding possible outcomes of Moral Case Deliberation, defined and perceived as important by the respondent. The answers on this question were used in the current study.

|  | **T.0 (N=384)** | **T.1 (N=232)** | **T.2 (N=53)** |
| --- | --- | --- | --- |
| Female N (%) | 236 (68) | 121 (67) | 28 (61) |
| Age mean (range) | 42 (22-65) | 44 (21-75) | 46 (25-65) |
| Years of experience mean (range) | 16 (0-44) | 16 (1-44) | 19 (1-44) |
| Profession N (%) |  |  |  |
| Nurse^1^ | 171 (45) | 67 (29) | 31 (59) |
| Doctor/specialist/psychiatrist | 25 (7) | 11 (5) | 0 |
| Therapist^2^ | 119 (31) | 110 (47) | 9 (17) |
| Manager^3^ | 24 (6) | 15 (7) | 4 (8) |
| Others^4^ | 35 (9) | 18 (8) | 1 (2) |
| Participants per setting N (%) |  |  |  |
| Community care services | 9 (2) | 4 (2) | 2 (4) |
| Somatic hospital care | 98 (26) | 35 (15) | 0 |
| Psychiatric care | 213 (56) | 148 (64) | 31 (59) |
| Mentally disabled care | 49 (13) | 26 (11) | 12 (23) |
| Health Inspection/Science | 15 (4) | 19 (8) | 8 (15) |
| Institutions N | 11 | 10 | 5 |
| ^1^Including registered nurses; assistant nurses, support workers and psychosocial workers,  ^2^Including physiotherapists; psychologists; spiritual caregivers; social workers  ^3^Including head of departments and policy makers,  ^4^Including volunteers, clients, researchers, trustees, secretary and interns | | | |
