# Supplementary material for: Defining and categorizing outcomes of Moral Case Deliberation (MCD): concept mapping with experienced MCD participants
Source: BMC Med Ethics. 2018 Nov 19;19:88. doi: 10.1186/s12910-018-0324-z (PMC6245560; doi:10.1186/s12910-018-0324-z)
Supplement: Supplementary file 2 — List of outcomes of Moral Case Deliberation. In this file, the list of 85 outcomes of Moral Case Deliberation is presented, as identified and defined by the participants during the brainstorm phase of the concept mapping focus group. (DOCX 17 kb) [file 12910_2018_324_MOESM2_ESM.docx]

**Additional file 2 – *List of outcomes of Moral Case Deliberation***

In this file, the list of 85 outcomes of Moral Case Deliberation is presented, as identified and defined by the participants during the brainstorm phase of the concept mapping focus group.

| ***English (translated by professional translator)*** |
| --- |
| 1. Substantiate decision made by staff member |
| 2. Feel you do not have to deal with the problem alone |
| 3. Support |
| 4. Support to proceed in a particular direction |
| 5. Accepting an outcome |
| 6. Curiosity about the other person |
| 7. Unity in teams/increased mutual cohesion |
| 8. Team spirit/ sense of belonging to group |
| 9. Difficult themes can become subject of discussion |
| 10. A concrete plan of action, a or b |
| 11. Plan of action on how to deal with damage |
| 12. Dilemma awareness, increased moral sensitivity |
| 13. More awareness of personal judgment |
| 14. Learn what the relevant norms and values are |
| 15. Knowledge of ethical concepts |
| 16. Understanding of interaction between norms and values |
| 17. Understanding which values and norms are in conflict with each other |
| 18. Understanding of diversity of norms and values, intrapersonal |
| 19. Understanding of diversity of norms and values, interpersonal |
| 20. More sensitive to the perspective of another person |
| 21. Accepting other person’s perspective |
| 22. Appreciate other person’s perspective |
| 23. Postpone personal judgments about individuals |
| 24. Postpone personal judgments about situations |
| 25. Examine personal judgment |
| 26. Identify relevant themes |
| 27. Identify recurring norms and values in particular themes |
| 28. Identify how moral issues are dealt with |
| 29. Reflexive skills |
| 30. Tools to reflect on moral dilemmas |
| 31. Clarify what good care entails |
| 32. Look for the answer for the client/central person in the case |
| 33. Relieves stress for case presenter |
| 34. Master at asking questions |
| 35. Contribute to organisational change or cultural shift |
| 36. Reduce blind spots |
| 37. Increase awareness of your blind spots |
| 38. Support in the development of new products and services |
| 39. Delight in astonishment about differences |
| 40. Less absence due to sickness |
| 41. Clarity about what the issue is and what is at stake |
| 42. More awareness of unequal balance of power |
| 43. Person presenting the case feels s/he is heard |
| 44. Team continues with method of examination as in moral case deliberation |
| 45. Team jointly determines team values |
| 46. Prevention of similar case/event in the future |
| 47. Anticipate, show restraint in similar case/event |
| 48. Initiate formulation of policy |
| 49. Disappointment about outcome |
| 50. Sense of wasting time |
| 51. More awareness how personal values influence working together |
| 52. Recognition brings sense of relief |
| 53. Sense of relief |
| 54. Fewer psychological complaints |
| 55. Less hierarchical interaction |
| 56. Gentler communication |
| 57. Increase range of action |
| 58. Creative thinking |
| 59. Enjoy your work more |
| 60. Get to know each other better |
| 61. Enhance professional identity |
| 62. Increased motivation regarding work |
| 63. Commitment to the organisation |
| 64. Help each other more readily |
| 65. Feel enriched by unexpected new insights |
| 66. You feel taken care of, nurturing for your inner self |
| 67. Be able to move on |
| 85. Understanding of role-related quality of norms and values |
| ***From Euro-MCD Instrument:*** |
| 68. More open communication |
| 69. Increased awareness of complexity of situation |
| 70. Greater opportunity for everyone to have their say |
| 71. Share difficult emotions and thoughts |
| 72. I listen more seriously to others’ opinions |
| 73. Increased awareness of own emotions |
| 74. Strengthened self-confidence |
| 75. Examine practice/policies |
| 76. Manage disagreements more constructively |
| 77. Gain more clarity about own responsibility |
| 78. Mutual respect |
| ***From open answers Euro-MCD Field study:*** |
| 79. Change your mind |
| 80. Confirmation of having made the right decision |
| 81. Take a step back to look at problem from a distance |
| 82. Better quality of work |
| 83. To feel safe |
| 84. Become more honest |
